# Supplementary material for: A conserved strategy of chalcone isomerase-like protein to rectify promiscuous chalcone synthase specificity
Source: Nat Commun. 2020 Feb 13;11:870. doi: 10.1038/s41467-020-14558-9 (PMC7018950; doi:10.1038/s41467-020-14558-9)
Supplement: Supplementary file 1 — Supplementary Information [file 41467_2020_14558_MOESM1_ESM.pdf]

A conserved strategy of chalcone isomerase-like protein to rectify promiscuous chalcone synthase specificity

Waki *et al.*

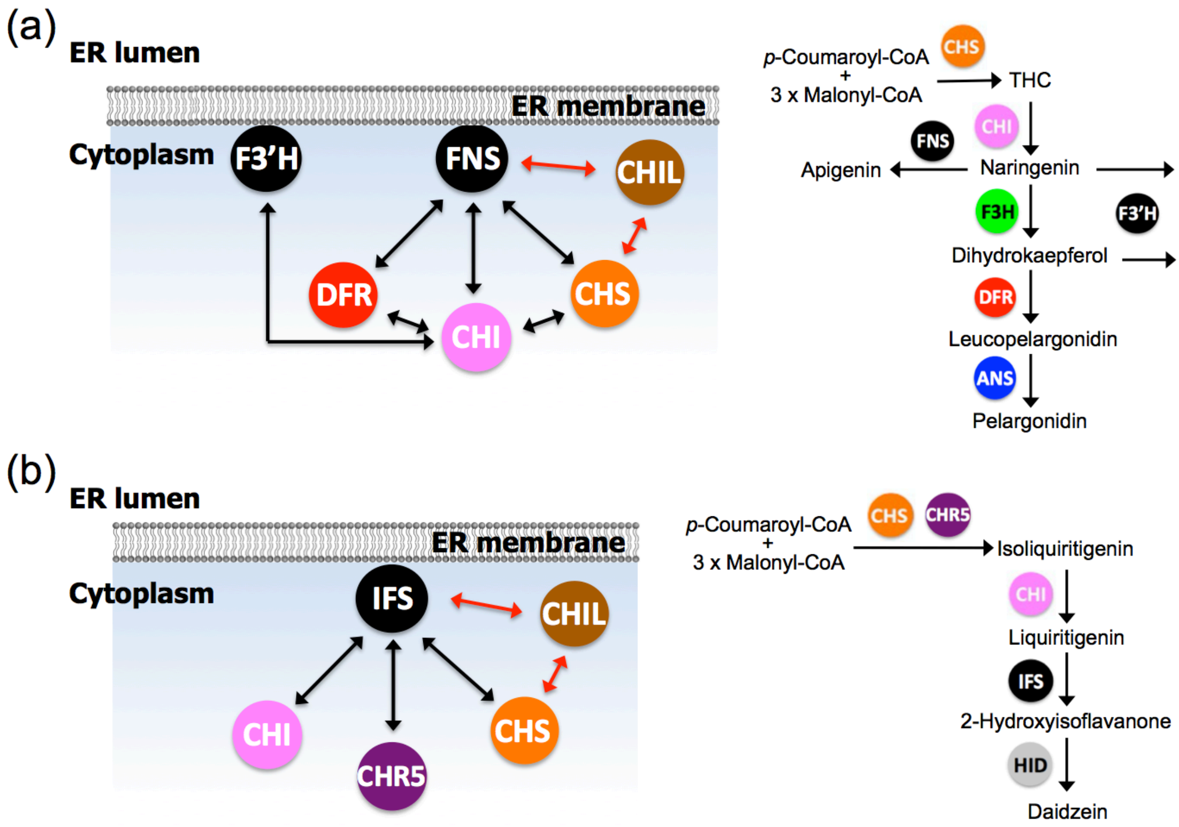

Supplementary Fig. 1. Protein-protein interactions among flavonoid enzymes. *Double-headed arrows* denote interactions between flavonoid enzymes (*circles*), with *black circles* indicating cytochromes P450 and *red double-headed arrows* indicating protein-protein interactions clarified in this study. (**a**) Interactions among flavonoid enzymes in snapdragon. *Right*, Proposed pathway of flavone and anthocyanin biosynthesis in snapdragon. CHIL, chalcone isomerase-like protein; CHS, chalcone synthase; CHI, chalcone isomerase; FNS, flavone synthase II; F3H, flavanone 3-hydroxylase; DFR, dihydroflavonol 4-reductase; ANS, anthocyanidin synthase; and F3'H, flavonoid 3'-hydroxylase. (**b**) Interactions among enzymes involved in daidzein biosynthesis in soybean. *Right*, Proposed pathway of daidzein biosynthesis in soybean. CHR5, the isozyme 5 of chalcone reductase; IFS, 2-hydroxyisoflavanone synthase; and HID, 2-hydroxyisoflavanone dehydratase.

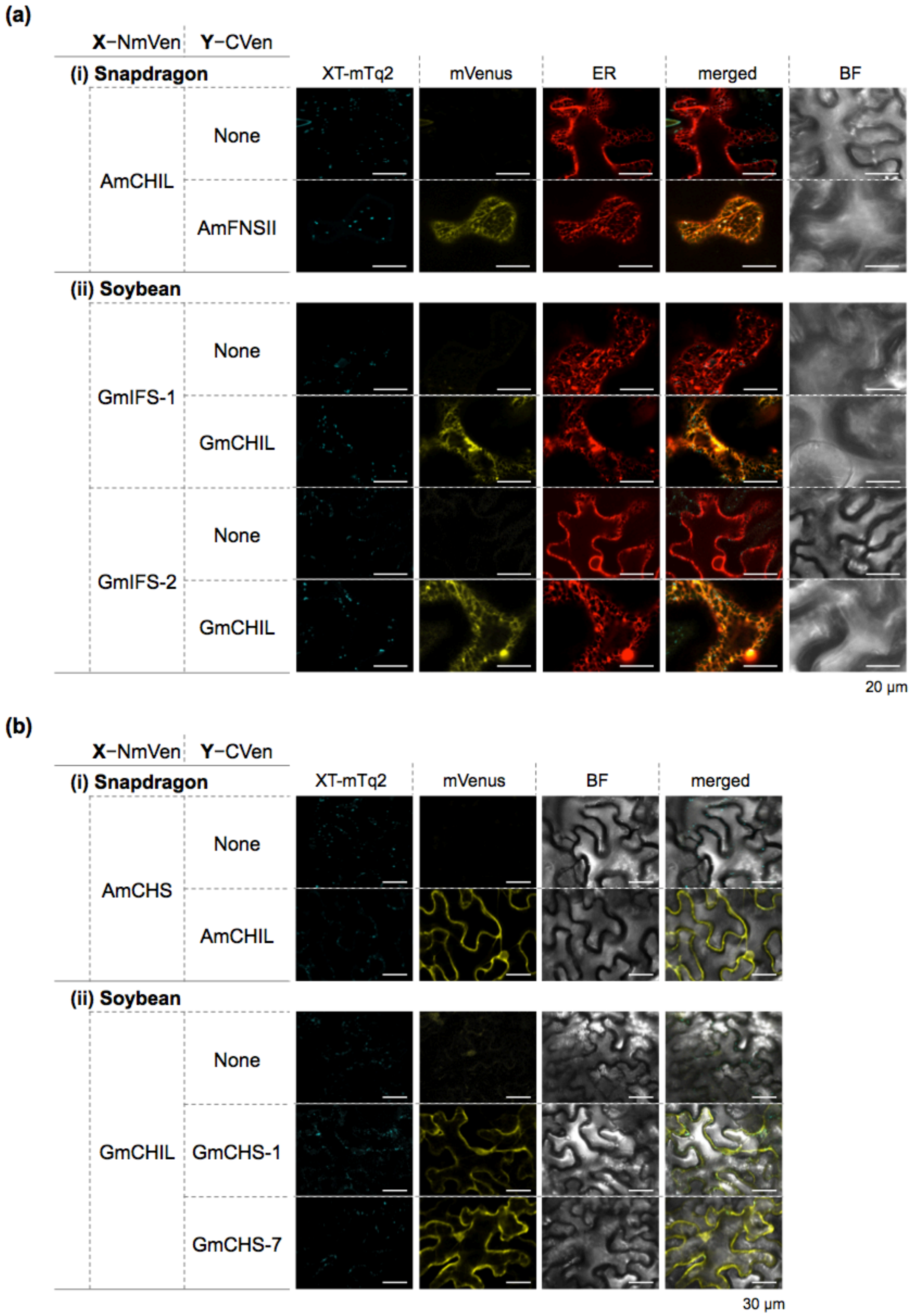

Supplementary Fig. 2. Detection of the binary interactions of CHIL with snapdragon and soybean flavonoid enzymes [(a) cytochromes P450 and (b) CHS] by BiFC. (**a**) (**i**) Detection of the binary interaction between CHIL and snapdragon flavone synthase II (AmFNSII). The AmCHIL-NmVen210, XT-mTq2 (a Golgi-localized marker), and mCherry-HDEL (an ER marker) constructs were co-expressed with CVen210 (*upper panels*) or AmFNSII-CVen210 (*lower panels*) in *Nicotiana benthamiana* leaf cells. (**ii**) Detection of the binary interaction between CHIL and soybean 2-hydroxyisoflavanone synthase (GmIFS) isozymes. The GmIFS-1-NmVen210 (or GmIFS-2-NmVen210), XT-mTq2, and mCherry-HDEL constructs were co-expressed with CVen210 (*upper panels*) or GmCHIL-CVen210 (*lower panels*) in *N. benthamiana* leaf cells. BF, transmitted-light images of cells. *Scale bars* = 20 μm. (**b**) (**i**) Detection of the binary interaction between CHIL and snapdragon CHS (AmCHS). The AmCHS-NmVen210 construct was co-expressed

with CVen210 (*upper panels*) or AmCHIL-CVen210 (*lower panels*) in *N. benthamiana* leaf cells. (**ii**) Detection of the binary interaction between CHIL and soybean CHS isozymes (GmCHS-1 and GmCHS-7). The GmCHIL-NmVen210 construct was co-expressed with CVen210 (*upper panels*), GmCHS-1-CVen210 (*middle panels*), or GmCHS-7-CVen210 (*lower panels*) in *N. benthamiana* leaf cells. BF, transmitted-light images of cells. *Scale bars* = 30 μm. Refer to the **Fig. 2** legend for details regarding the protein names.

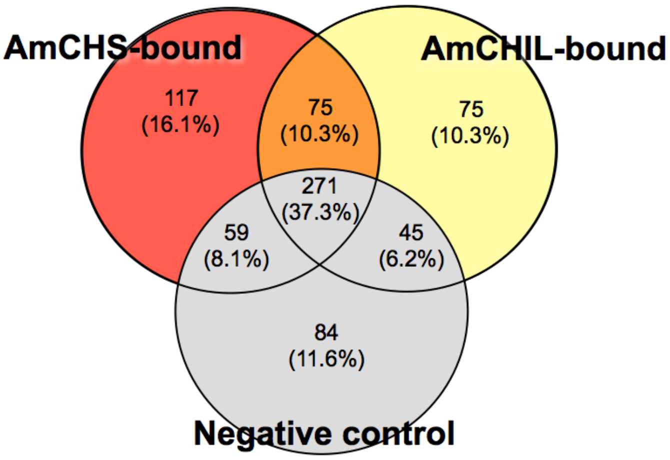

Supplementary Fig. 3. Venn diagram of the results of LC-MS/MS analysis that followed co-precipitation experiments. The Negative control circle indicates the number of protein species bound to Ni^{2+}-coated beads. The AmCHS-bound circle indicates the number of protein species bound to His_6-AmCHS-bound Ni^{2+}-coated beads. The AmCHIL-bound circle indicates the number of protein species bound to His_6-AmCHIL-bound Ni^{2+}-coated beads. The numbers in the red and yellow portions of the diagram (117 and 75) refer to the number of protein species that were specifically bound to His_6-AmCHS-bound- and His_6-AmCHIL-bound beads, which included AmCHIL (Am07g21400.P01) and AmCHS (Am04g40840.P01), respectively. The number in the orange portion of the diagram (75) represents the number of protein species that were bound to both His_6-AmCHS-bound and His_6-AmCHIL-bound beads.

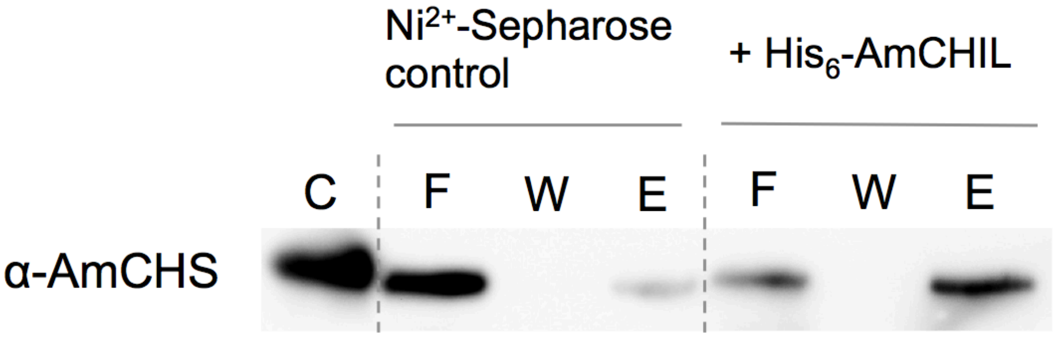

Supplementary Fig. 4. Co-precipitation experiments followed by immunoblot analysis. His_6-AmCHIL was added to the extract from red snapdragon petals. Ni^{2+}-coated Sepharose beads were then added to the mixture and incubated at 4°C for 1 h, followed by centrifugation. The supernatant (termed fraction F) was recovered. The beads were washed three times with 0.05 M HEPES-NaOH, pH 7.5 and the third-wash supernatant (termed fraction W) was recovered. The bead-bound proteins were then eluted by washing the beads with the buffer containing 500 mM imidazole (termed fraction E). SDS-PAGE and western blotting analyses were carried out using anti-AmCHS IgG as the primary antibody. For the control (Ni^{2+}-Sepharose control), His_6-AmCHIL was replaced by water. C, The extract from red snapdragon petals. For further experimental details, see **Methods**.

The results of the control experiment showed non-specific binding of a small amount of AmCHS to Ni^{2+}-coated Sepharose beads. However, with addition of His_6-AmCHIL, the amount of AmCHS in fraction F greatly diminished and that of the bead-bound AmCHS was increased (fraction E). These results show the specific binding of AmCHS to AmCHIL in the extract from snapdragon petals. The source data of the immunoblots are provided in the Source Data file.

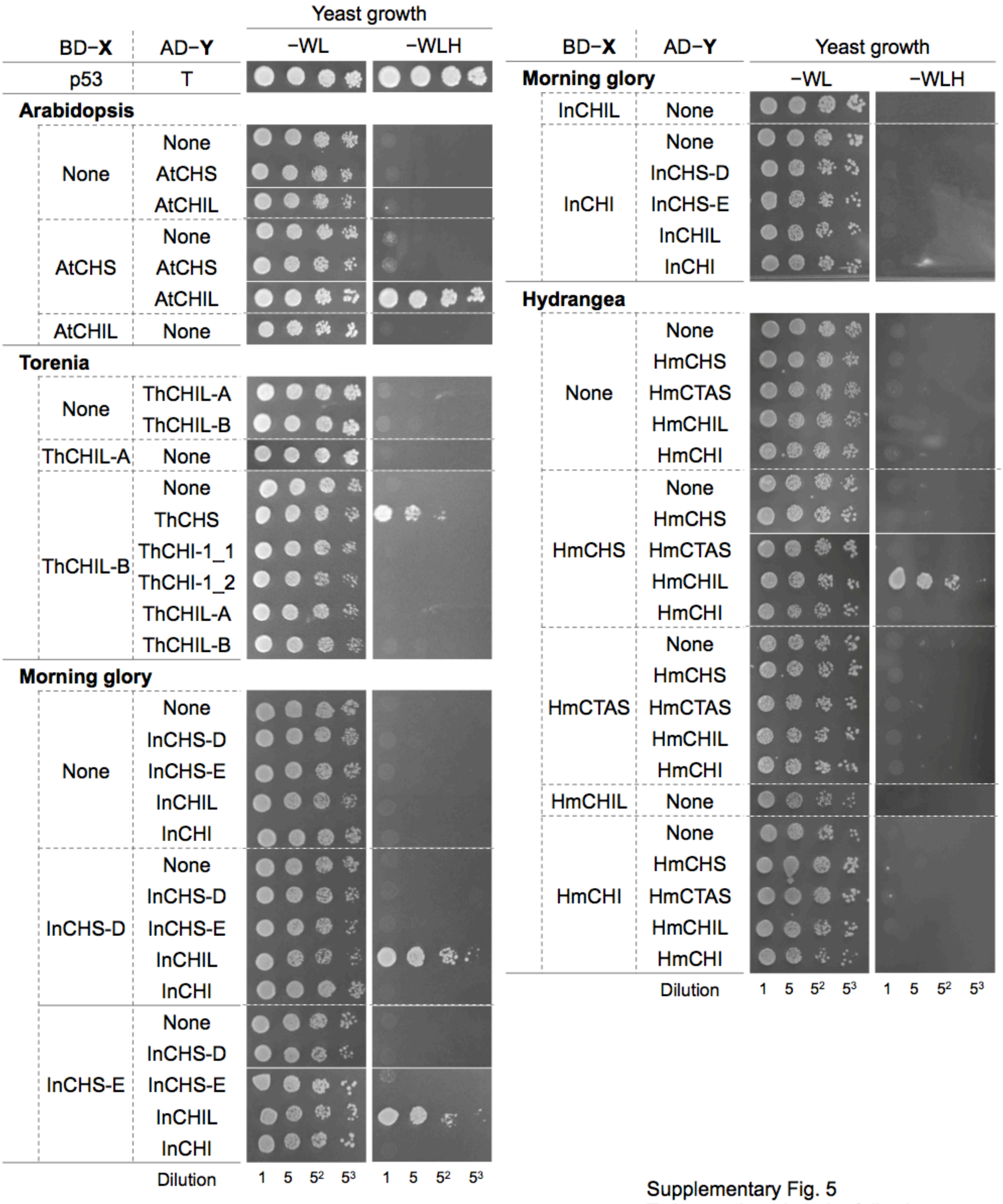

Supplementary Fig. 5
To be continued on the following page

Continuation of Supplementary Fig. 5

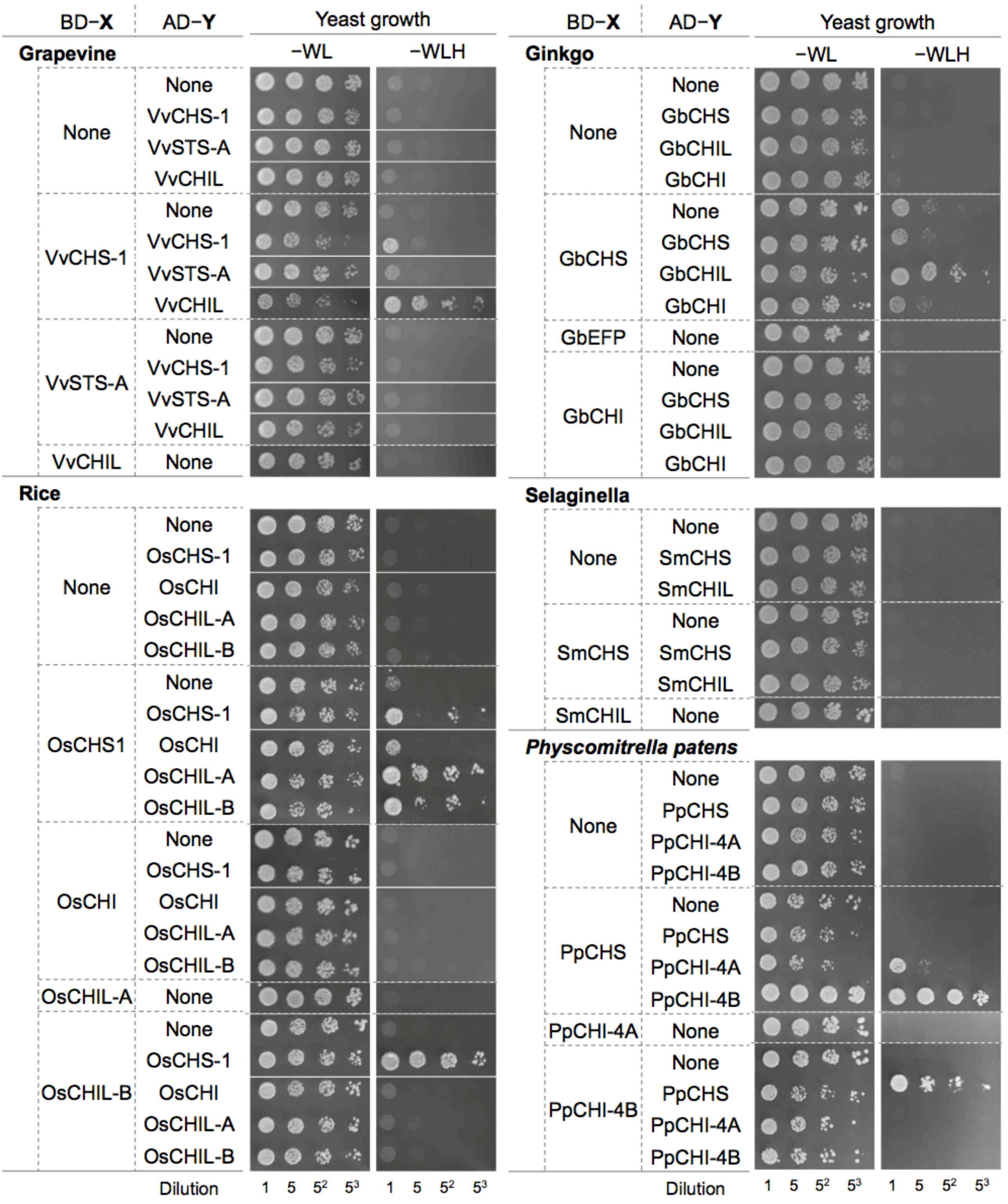

Supplementary Fig. 5. Analyses of the binary interactions among CHIL, CHS, and CHI from various land plant species in a yeast two-hybrid system. The growth of yeast cells co-expressing CHIL, CHS, and CHI fused to the activation domain (AD, AD-Y column) and the DNA-binding domain (BD, BD-X column) of the yeast GAL4 transcription factor is presented. Refer to the **Fig. 2** legend for details regarding the protein names. “None” in the BD-X and AD-Y columns refers to yeast cells co-expressing BD and AD (without a fused protein), respectively. Abbreviated growth media names are as follows: −WL, SD agar medium lacking tryptophan and leucine; −WLH, −WL medium lacking histidine.

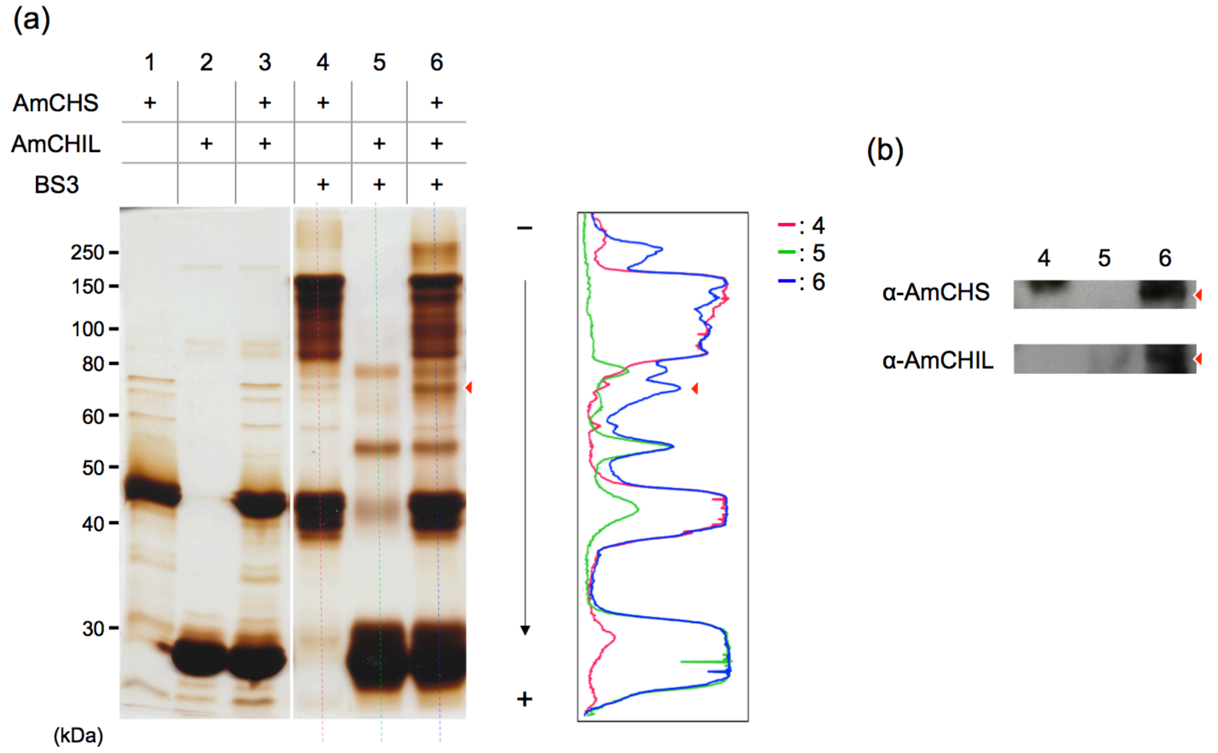

Supplementary Fig. 6. Stoichiometric analysis of CHIL binding to CHS. (a) A mixture containing His_6-AmCHIL and His_6-AmCHS was reacted with BS3 at pH 7.5 for 5 min, followed by SDS-PAGE (*lane* 6). For controls, one or two of these components were omitted (*lanes* 1 through 5). Proteins in the gels were visualized by silver staining (see below). The *arrow* indicates the direction of electrophoresis. *Right panel*, densitometric traces of *lanes* 4, 5, and 6 (scanned and represented by *red, green,* and *blue dotted lines*, respectively). *Red arrow heads* indicate the unique protein band with a molecular mass of 70 kDa in the BS3-reacted protein mixture. (b) The results of immunoblot analysis of *lanes 4* through *6* showing that the 70-kDa protein band (indicated by *red arrow heads* in (a)) was immuno-reactive to anti-AmCHS IgG (*top*) and anti-AmCHIL IgG (*bottom*). It must be mentioned that the 72-kDa band in *lane* 4 was found to be immuno-reactive to anti-AmCHS IgG. This band likely arose from a very small amount of His_6-AmCHS that was cross-linked with a contaminating *E. coli* protein and was visualized by the present high-sensitive immunoblot analysis. Contaminating *E. coli* proteins in the gels were not detected by staining with Coomassie Brilliant Blue R250 but could only be visualized after silver staining. The source data of the immunoblots are provided in the Source Data file.

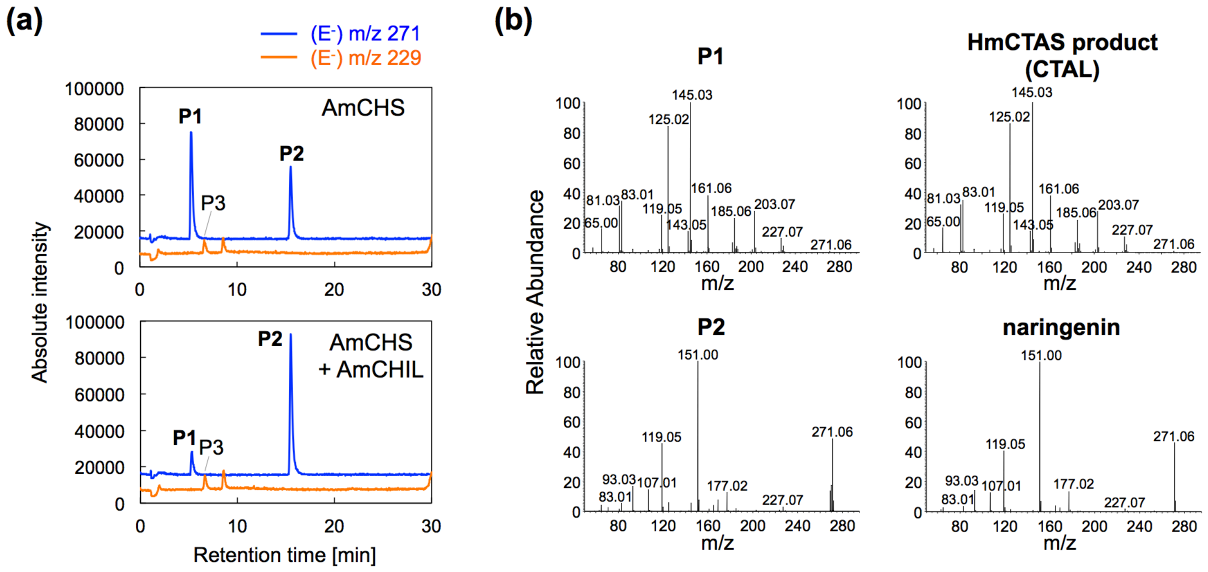

Supplementary Fig. 7. Analyses of the products of the AmCHS-catalyzed reaction with *p*-coumaroyl-CoA and malonyl-CoA in the presence and absence of AmCHIL. (**a**) HPLC chromatograms of the products of the AmCHS-catalyzed reaction with *p*-coumaroyl-CoA and malonyl-CoA in the presence (*lower panel*) and absence (*upper panel*) of AmCHIL. Chromatograms were monitored by mass spectrometry at *m/z* of 271 (*blue line*) and 229 (*orange line*). The THC produced via the CHS-catalyzed reaction was non-enzymatically and fully converted to naringenin under the experimental conditions. (**b**) The MS spectra of peaks 1 and 2 (P1 and P2) in panel (**a**) were analyzed by tandem MS spectrometry (*left*) and compared with those of CTAL prepared via the HmCTAS-catalyzed reaction with *p*-coumaroyl-CoA and malonyl-CoA (*upper right*) and naringenin (*lower right*).

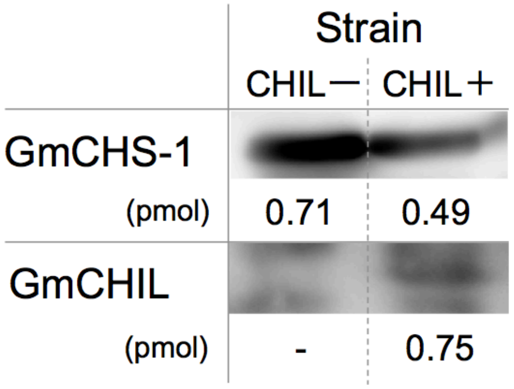

Supplementary Fig. 8. Immunological quantification of GmCHS-1 and GmCHIL proteins that were produced in the engineered *E. coli* cells (CHIL– and CHIL+). For experimental details, see **Methods**. The source data of the immunoblots are provided in the Source Data file.

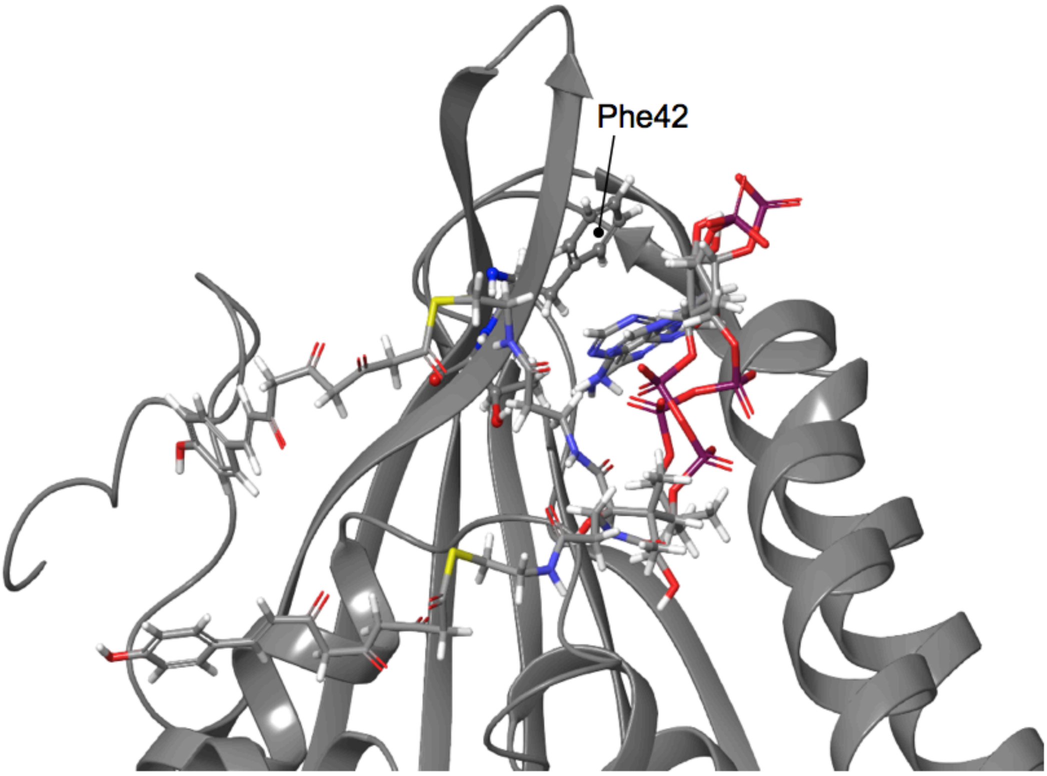

Supplementary Fig. 9. Top two modeling results for AtCHIL (PDB ID: 4DOK; *gray ribbon*) with a docked *p*-coumaroyl-tetraketide-CoA ligand (*sticks*). Both the Prime/MM-GBSA and the chemPLP calculations, which estimated the affinity and binding energy between *p*-coumaroyl-tetraketide-CoA and the AtCHIL crystal structure, produced the top two docking results. These results are very similar regarding the CoA portion of *p*-coumaroyl-tetraketide-CoA, where the adenine moiety of the CoA is inserted into the deep pocket and is stacked with the ring of Phe42. The rest of the ligand molecule is either wrapped around the surface of the β-sheet (upper molecule, *sticks*) or is placed in the groove created by the two α-helices (lower molecule, *sticks*). Regardless, both docking variants have a very similar estimated ΔG of binding (−61.22 kcal/mol and −61.33 kcal/mol, respectively). In contrast, small-molecule ligands, which are co-crystallized with the known structures submitted to the Protein Data Bank, have a ΔG of binding between −30 kcal/mol and −120 kcal/mol. Thus, the binding of *p*-coumaroyl-tetraketide-CoA to AtCHIL as shown here is possible and energetically favorable.

(a)

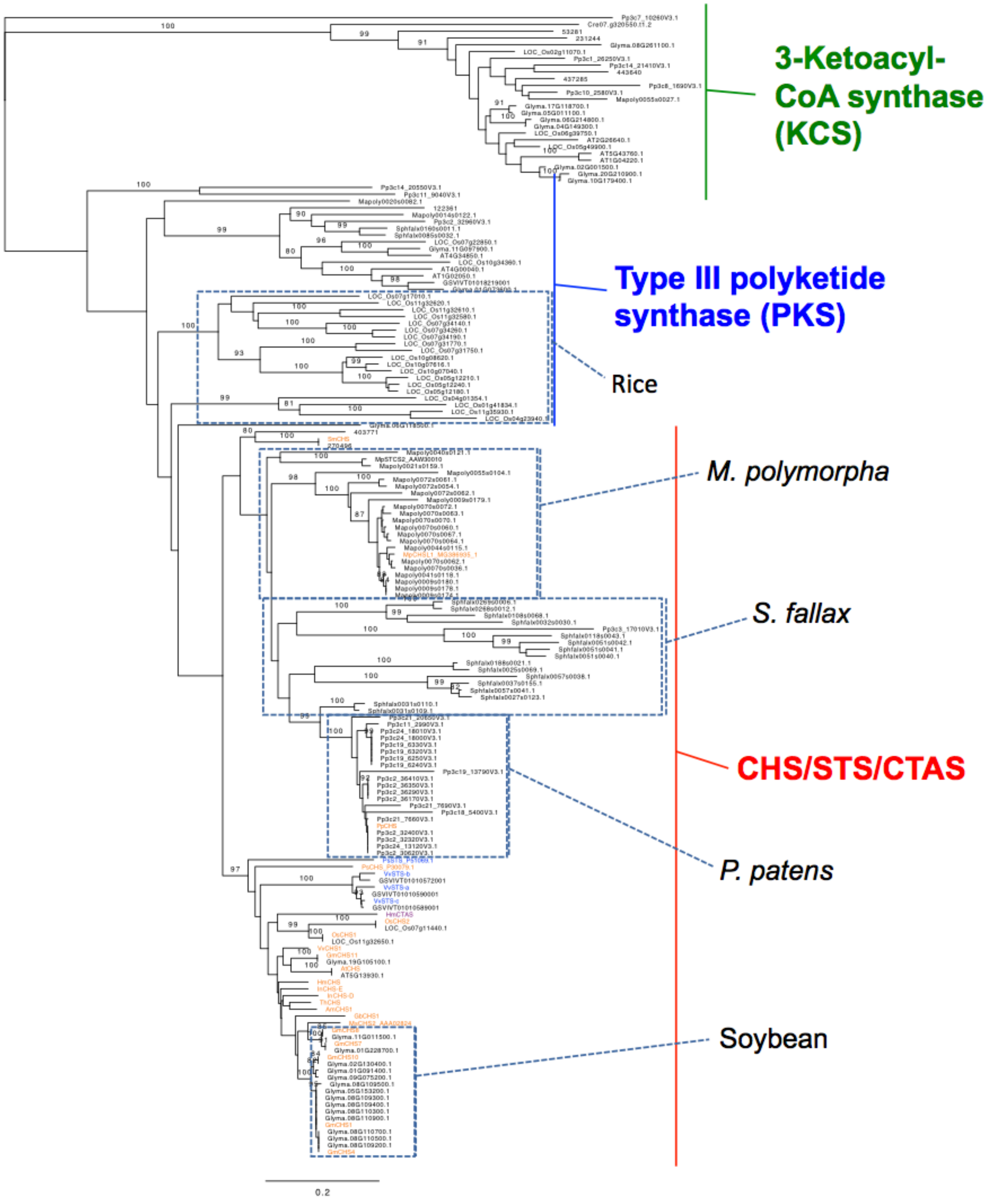

Supplementary Fig. 10
to be continued on the following page

(Continuation of Supplementary Fig. 10)

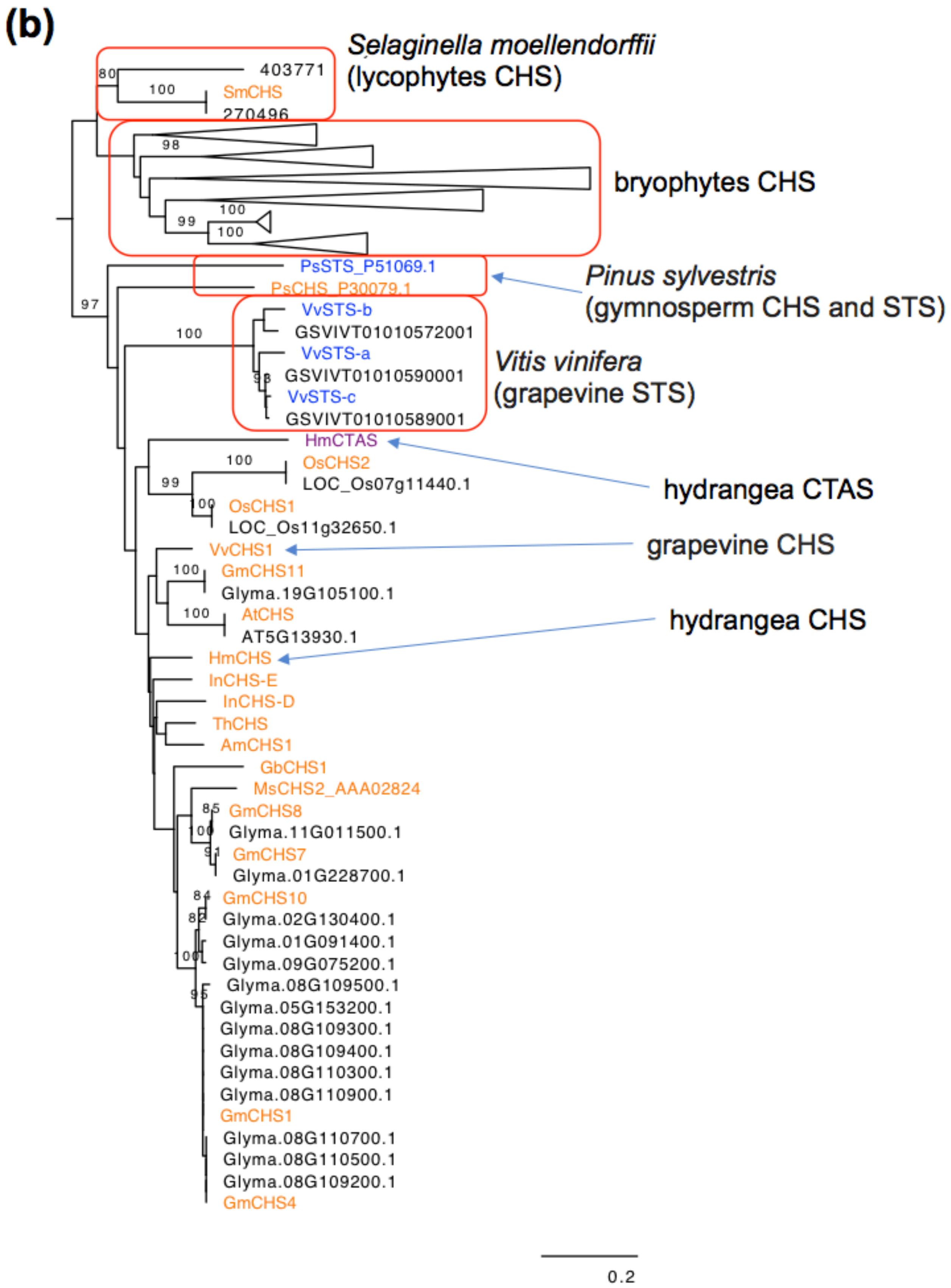

Supplementary Fig. 10
to be continued on the following page

(Continuation of Supplementary Fig. 10)

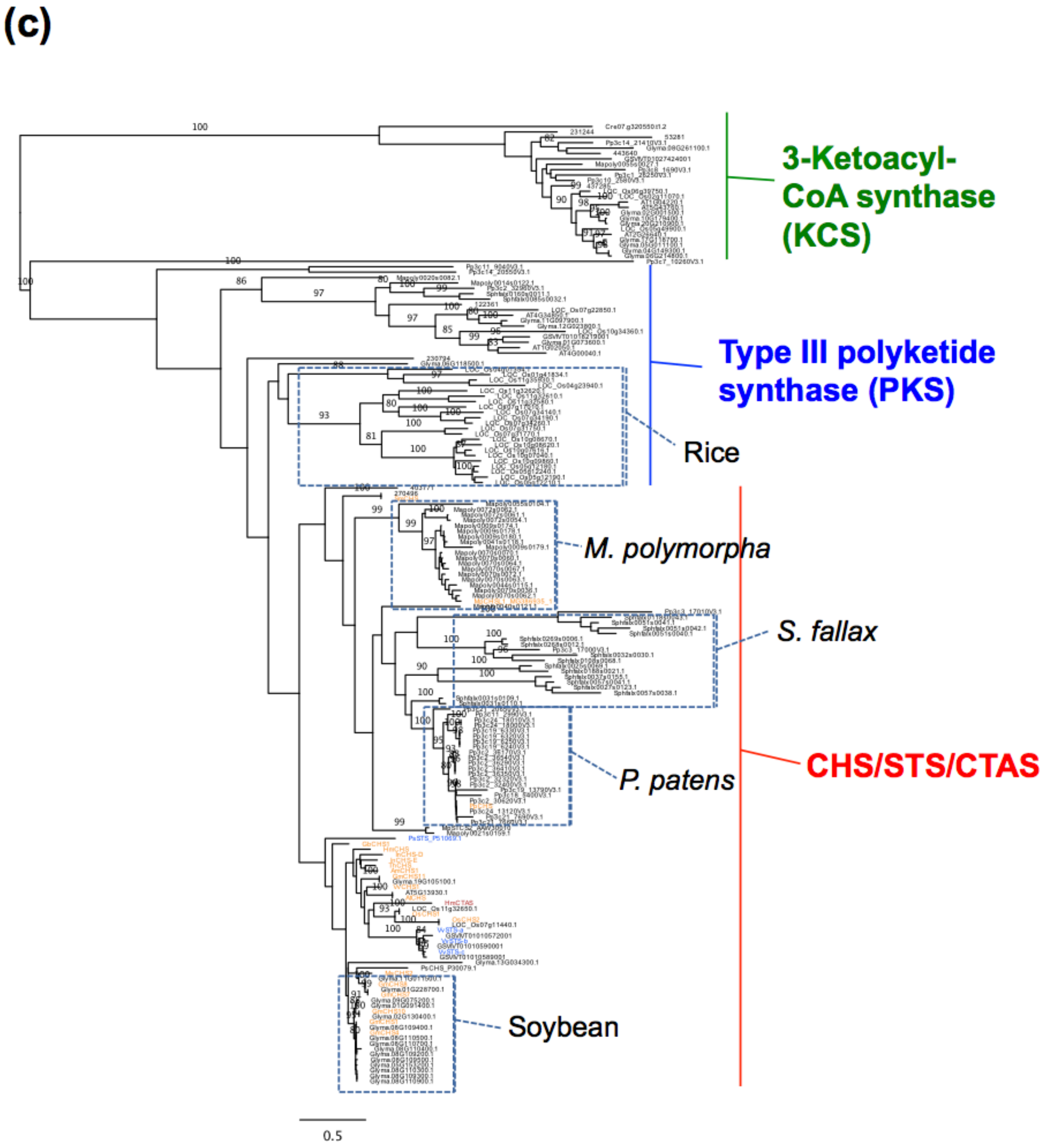

Supplementary Fig. 10
to be continued on the following page

(Continuation of Supplementary Fig. 10)

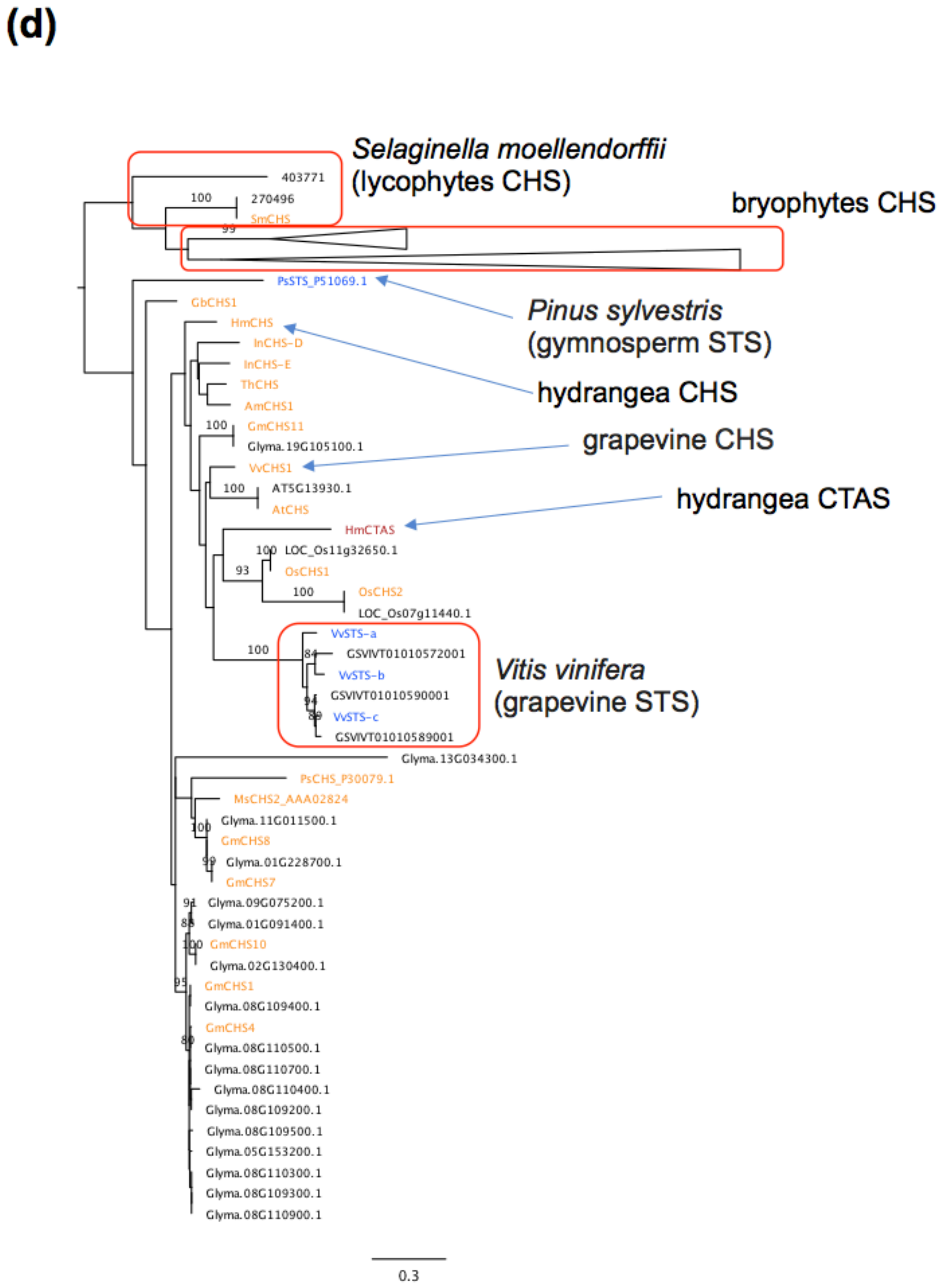

Supplementary Fig. 10
to be continued on the following page

Supplementary Fig. 8. Phylogenetic trees of CHS and related proteins. Phylogenetic analyses were performed using the neighbor-joining (NJ) method ((**a**) and (**b**)) and the maximum likelihood (ML) method ((**c**) and (**d**)). Sequence names shown in color indicate the enzymes that were functionally characterized (*orange*, CHS; *magenta*, CTAS; *blue*, STS). Confidence of inferred phylogenetic trees were assessed by bootstrap method [48] with 100 replicates and bootstrap values are indicated beside branches. Bar = (a) 0.2, (b) 0.2, (c) 0.5, and (d) 0.3 amino acid substitutions per site. Consistent tree topologies were obtained with both phylogenetic methods.

(**a**) and (**c**) Phylogenetic trees of CHSs, STSs, and CTASs (indicated by *vertical red line*) as well as their remotely homologous proteins [3-ketoacyl-CoA synthases (*vertical green line*) and PKSs (*vertical blue line*)]. A PKS-related multigene family of rice and CHS/STS/CTAS-related multigene families of *M. polymorpha*, *S. fallax*, *P. patens*, and soybean are *boxed* with *dotted lines*.

(**b**) and (**d**) Subtrees of CHS, STS, and CTAS from the phylogenetic trees shown in (**a**) and (**c**), respectively, are magnified. Multigene families of bryophyte CHS, *Vitis vinifera* CHS, and *S. moellendorffii* CHS are *boxed* with *red lines*.

Supplementary Table 1. Kinetic and thermodynamic properties of binary interactions between CHIL and CHS of some plant species as determined by biolayer interferometry.

Phylogenetics	Plant	CHS	CHIL	k_a [$M^{-1}s^{-1}$]	k_d [s^{-1}]	K_D (nM)
Bryophyte	*P. patens*	PpCHS	PpCHIL-A	1.18×10^{5}	3.19×10^{-4}	2.69
Lycophyte	*S. moellendorffii*	SmCHS	SmCHIL	1.19×10^{4}	6.74×10^{-4}	56.6
Gymnosperm	*G. biloba*	GbCHS	GbCHIL	2.02×10^{4}	1.54×10^{-4}	126
Angiosperm						
Monocot	Rice	OsCHS-1	OsCHIL-A	1.04×10^{4}	6.72×10^{-4}	64.4
Dicot	Soybean	GmCHS-1	GmCHIL	5.10×10^{4}	3.81×10^{-4}	7.46
		GmCHS-7	GmCHIL	5.83×10^{4}	1.13×10^{-3}	19.3
	Arabidopsis	AtCHS	AtCHIL	1.50×10^{5}	1.37×10^{-3}	9.15
	Hydrangea	HmCHS	HmCHIL	1.15×10^{5}	2.77×10^{-3}	24.1
		HmCTAS	HmCHIL	N. D.	N. D.	
	Morning glory	InCHS-D	InCHIL	1.96×10^{5}	2.06×10^{-4}	1.05
	Snapdragon	AmCHS	AmCHIL	2.93×10^{4}	1.83×10^{-3}	62.6

N. D., Binding not detected.

Supplementary Table 2. Primers used for complementation experiments with *A. thaliana* T-DNA insertion mutants

Primer name / description	Nucleotide sequence
CHIL-F / At5g05270_2F	5'-GTAAGTCTTACACTCTTTGC-3'
CHIL-R / At5g05270_1553R	5'-CTATTCTGTGTCCTTCCAATGTTGA-3'
TF / pAC161LB 8761	5'-GGGCTACACTGAATTGGTAGCTC-3'
TR / pAC161RB 2591	5'-CAGGGTTTTCCCAGTCACGACG-3'
AF / At5g05270+CACC1696F	5'-CACCATCTCAATTTGGAGAAATTAT-3'
AR / At5g05270-25R	5'-TTCTAATTTATATTATTTTGAATCTG-3'
BF / At3g55120infAt5g05270F	5'-TAATATAAATTAGAAATGTCTTCATCCAACGCC-3'
BR / At3g55120CDSinsertR2	5'-GGCGCGCCCACCCTTCCTAAACTTTATTATA-3'
CF / At5g05270infAt5g05270F	5'-TAATATAAATTAGAAATGGGAACAGAGATGGTC-3'
CR / At5g05270CDSinsertR2	5'-GGCGCGCCCACCCTTCTGAATCCAAACGAGC-3'
DF / No105_pdr11242insertF	5'-TAATATAAATTAGAAATGGGTCCCCAAGTT-3'
DR / No105_pdr11242insertR	5'-GGCGCGCCCACCCTTGGCAGCAATGGCTTC-3'
EF / No104_pdp75026insertF	5'-TAATATAAATTAGAAATGGGTCTCCAAGTT-3'
ER / No104_pdp75026insertR	5'-GGCGCGCCCACCCTTTGCAACCTGGGCAGC-3'
FF / Ipomea nil_EFPinserF	5'-TAATATAAATTAGAAATGGGTACTGAAATG-3'
FR / Ipomea nil_EFPinserR	5'-GGCGCGCCCACCCTTACACAAAGGGAACAT-3'
GF / m_polymorphal_EFPinsertF	5'-TAATATAAATTAGAAATGGCTCAAGGCGAC-3'
GR / m_polymorphal_EFPinsertR	5'-GGCGCGCCCACCCTTTCATGCCAGTAAGGC-3'
HF / m_polymorphal_CHIinsertF	5'-TAATATAAATTAGAAATGGAGTCTACGCAT-3'
HR / m_polymorphal_CHIinsertR	5'-GGCGCGCCCACCCTTCTAAGATGCCCCGTT-3'
AtCHI_RT_F	5'-AGCTTCATGTAGACTCCGT-3'
AtCHI_RT_R	5'-CGTCCTTGTTCTTCATCATT-3'
At5g05270_359F	5'-GAGATCCACTTTCTTCAAGT-3'
AtCHIL_RT_R	5'-AAACTGCGGAGATTGAATCA-3'
Ipom_EFP_RT_F	5'-TGGTGATGGTGGATGAAATC-3'
Ipom_EFP_RT_R	5'-ATTCACTTAGTCAGGCAGTA-3'
EFPa105_RT_F	5'-AGTTGAAGACATCGACTTTG-3'
EFPa105_RT_R	5'-AAGAGTGGAGGGCGTGATA-3'
EFPb104_RT_F	5'-ATGTAGAAGGCATCGACTTC-3'
EFPb104_RT_R	5'-TGTTCTCGCCCAAGTAAAG-3'
poly_EFP_RT_F	5'-ACTATGCCGTGGATGGTAT-3'
poly_EFP_RT_R	5'-CTGATGTAGTCTCCGACATT-3'
poly_CHI_RT_F	5'-TACGCATTCGAAAGTGACTT-3'
poly_CHI_RT_R	5'-AGACATGTTTTCGCCAAACT-3'

Supplementary Table 3. Plasmids used for complementation experiments with *A. thaliana* T-DNA insertion mutants

Plasmid	Description
pKYS454	pENTR/D-TOPO/1696-bp fragment of AtCHIL promoter region fused to AtCHI CDS
pKYS455	pENTR/D-TOPO/1696-bp fragment of AtCHIL promoter region fused to AtCHIL CDS
pKYS459	pENTR/D-TOPO/1696bp fragment of AtCHIL promoter region fused to PpCHIL-A CDS
pKYS458	pENTR/D-TOPO/1696bp fragment of AtCHIL promoter region fused to PpCHIL-B CDS
pKYS462	pENTR/D-TOPO/1696bp fragment of AtCHIL promoter region fused to InCHIL CDS
pKYS463	pENTR/D-TOPO/1696bp fragment of AtCHIL promoter region fused to MpCHIL CDS
pKYS464	pENTR/D-TOPO/1696bp fragment of AtCHIL promoter region fused to MpCHI CDS

Supplementary Table 4. cDNAs of flavonoid enzymes and related proteins used in this study

Enzyme/protein	Gene	Accession number*1, locus name*2, or contig name*3, *4, *5
Physcomitrella patens		
Chalcone synthase	*PpCHS*	Pp3c24_13120*2
Chalcone isomerase-like	*PpCHIL-A*	Pp3c4_25770*2
	PpCHIL-B	Pp3c426_4040*2
Marchantia polymorpha		
Chalcone isomerase	*MpCHI*	Mapoly0167s0012*3
Chalcone isomerase-like	*MpCHIL*	Mapoly0175s0004*3
Selaginella (*Selaginella moellendorffii*)		
Chalcone synthase	*SmCHS*	270496*2
Chalcone isomerase-like	*SmCHIL*	227414*2
Ginkgo (*Ginkgo biloba*)		
Chalcone synthase	*GbCHS*	AY647263*1
Chalcone isomerase	*GbCHI*	07234*4
Chalcone isomerase-like	*GbCHIL*	07706*4
Rice (*Oryza sativa*)		

Chalcone synthase	*OsCHS-1*	LOC_Os11g32650*2
Chalcone isomerase	*OsCHI*	LOC_Os03g60509*2
Chalcone isomerase-like	*OsCHIL-A*	LOC_Os11g02440*2
	OsCHIL-B	LOC_Os12g02370.2*2
Grapevine (*Vitis vinifera*)		
Chalcone synthase	*VvCHS*	GSVIVT01032968001*2
Stilbene synthase	*VvSTS*	XM_002271335*1
Chalcone isomerase	*VvCHI*	GSVIVT01032619001*2
Chalcone isomerase-like	*VvCHIL*	GSVIVT01032685001*2
Soybean (*Glycine max* (L.) Merr.)		
4-coumarate:coenzyme A ligase	*Gm4CL-3*	AF002258*1
Chalcone synthase	*GmCHS-1*	X54644, Glyma.08G109400*2
	GmCHS-7	M98871, Glyma.01G228700*2
Chalcone reductase	*GmCHR-1*	X55730, Glyma.14G005700*2
	GmCHR-5	LC309095, Glyma.18G285800*2
Chalcone isomerase	*GmCHI-2*	AY595415, Glyma.20G241700*2
	GmCHI-1A	AY595413, Glyma.20G241500*2
	GmCHI-1B2	AY595419, Glyma.10G292200*2
Chalcone isomerase-like	*GmCHIL*	AAT94362, Glyma.06G143000*2
2-Hydroxyisoflavanone synthase	*GmIFS-1*	AF195818, Glyma.07G202300*2
	GmIFS-2	AF195819, Glyma.13G173500*2
2-Hydroxyisoflavanone dehydratase	*GmHID-1*	AB154415, Glyma.01G239600*2

Arabidopsis (*Arabidopsis thaliana*)		
Chalcone synthase	*AtCHS (TT4)*	AT5G13930[*2]
Chalcone isomerase	*AtCHI (TT5)*	AT3G55120[*2]
Chalcone isomerase-like	*AtCHIL*	AT5G05270[*2]
Hydrangea (*Hydrangea macrophylla*)		
Chalcone synthase	*HmCHS*	AB011467[*1]
Coumaroyl triacetic acid synthase	*HmCTAS*	AB011468[*1]
Chalcone isomerase	*HmCHI*	Hydrangea_macrophylla_12062016_isotig16454[*5]
Chalcone isomerase-like	*HmCHIL*	Hydrangea_macrophylla_12062016_isotig23148[*5]
Morning glory (*Ipomoea nil*)		
Chalcone synthase	*InCHS-D*	AB001818[*1]
	InCHS-E	AB001819[*1]
Chalcone isomerase	*InCHI*	XM_019337142[*1]
Chalcone isomerase-like	*InCHIL*	AB545800[*1]
Snapdragon (*Antirrhinum majus* L.)		
Chalcone synthase	*AmCHS*	X03710[*1]
Chalcone isomerase	*AmCHI*	AB861648[*1]
Chalcone isomerase-like	*AmCHIL*	AB861649[*1]
Flavone synthase II	*AmFNSII*	AB028151[*1]
Flavanone 3-hydroxylase	*AmF3H*	LC194907[*1]

Dihydroflavonol 4-reductase	*AmDFR*	P14721[*1]
Flavonoid 3’-hydroxylase	*AmF3'H*	DQ272592[*1]
Torenia (*Torenia hybrida*)		
Chalcone synthase	*ThCHS*	AB012923[*1]
Chalcone isomerase	*ThCHI1-1*	LC194908[*1]
	ThCHI1-2	LC194909[*1]
Chalcone isomerase-like	*ThCHIL-A*	AB543055[*1]
	ThCHIL-B	AB543056[*1]

[*1] DDBJ/EMBL/GenBank accession number.

[*2] Phytozome v12.1 locus name. https://phytozome.jgi.doe.gov/pz/portal.html

[*3] MarpolBase contig name. http://marchantia.info

[*4] GigaDB contig name. http://gigadb.org/dataset/100209

[*5] Hardwood Genomics Project contig name. https://www.hardwoodgenomics.org/
