# Supplementary material for: A conserved strategy of chalcone isomerase-like protein to rectify promiscuous chalcone synthase specificity
Source: Nat Commun. 2020 Feb 13;11:870. doi: 10.1038/s41467-020-14558-9 (PMC7018950; doi:10.1038/s41467-020-14558-9)
Supplement: Supplementary file 3 — Reporting Summary [file 41467_2020_14558_MOESM3_ESM.pdf]

nature research

Corresponding author(s): Toru Nakayama

Last updated by author(s): 2019/12/09

Reporting Summary

Nature Research wishes to improve the reproducibility of the work that we publish. This form provides structure for consistency and transparency in reporting. For further information on Nature Research policies, see Authors & Referees and the Editorial Policy Checklist .

Statistics

For all statistical analyses, confirm that the following items are present in the figure legend, table legend, main text, or Methods section.

n/a	Confirmed	
☐	☒	The exact sample size (*n*) for each experimental group/condition, given as a discrete number and unit of measurement
☐	☒	A statement on whether measurements were taken from distinct samples or whether the same sample was measured repeatedly
☐	☒	The statistical test(s) used AND whether they are one- or two-sided<br>*Only common tests should be described solely by name; describe more complex techniques in the Methods section.*
☒	☐	A description of all covariates tested
☒	☐	A description of any assumptions or corrections, such as tests of normality and adjustment for multiple comparisons
☐	☒	A full description of the statistical parameters including central tendency (e.g. means) or other basic estimates (e.g. regression coefficient) AND variation (e.g. standard deviation) or associated estimates of uncertainty (e.g. confidence intervals)
☒	☐	For null hypothesis testing, the test statistic (e.g. *F*, *t*, *r*) with confidence intervals, effect sizes, degrees of freedom and *P* value noted<br>*Give P values as exact values whenever suitable.*
☒	☐	For Bayesian analysis, information on the choice of priors and Markov chain Monte Carlo settings
☒	☐	For hierarchical and complex designs, identification of the appropriate level for tests and full reporting of outcomes
☒	☐	Estimates of effect sizes (e.g. Cohen's *d*, Pearson's *r*), indicating how they were calculated

Our web collection on statistics for biologists contains articles on many of the points above.

Software and code

Policy information about availability of computer code

Data collection	n/a
Data analysis	The SigmaPlot 12 program, which is commercially available from Hulinks, Tokyo, Japan, was used for kinetic analyses.

For manuscripts utilizing custom algorithms or software that are central to the research but not yet described in published literature, software must be made available to editors/reviewers. We strongly encourage code deposition in a community repository (e.g. GitHub). See the Nature Research guidelines for submitting code & software for further information.

Data

Policy information about availability of data

All manuscripts must include a data availability statement. This statement should provide the following information, where applicable:

- Accession codes, unique identifiers, or web links for publicly available datasets
- A list of figures that have associated raw data
- A description of any restrictions on data availability

The following statement has been made in 'Data availability' section: All data needed to evaluate the conclusions in the manuscript are presented herein and/or as supplementary materials. Additional data related to this study may be requested from the authors. The sequences for all genes described in this manuscript are available in the GenBank/EMBL/DDBJ databases and other databases shown in Supplementary Table 4. The source data underlying Figs. 4, 5, and 6 as well as the source data of the gels and immunoblots in Supplementary Figs. 4, 6b, and 8 are provided in the Source Data file.

Field-specific reporting

Please select the one below that is the best fit for your research. If you are not sure, read the appropriate sections before making your selection.

Life sciences study design

All studies must disclose on these points even when the disclosure is negative.

Sample size	Data are presented as the average of three or more independent determinations (± standard deviation).
Data exclusions	No data were excluded from the analyses.
Replication	Data are presented as the average of three or more independent determinations (± standard deviation).
Randomization	Because there were no need to allocate samples into experimental groups in essence, this is not relevant to our study.
Blinding	Because there were no need to allocate samples into experimental groups in essence, this is not relevant to our study.

Reporting for specific materials, systems and methods

We require information from authors about some types of materials, experimental systems and methods used in many studies. Here, indicate whether each material, system or method listed is relevant to your study. If you are not sure if a list item applies to your research, read the appropriate section before selecting a response.

Materials & experimental systems

n/a	Involved in the study	
☐	☒	Antibodies
☒	☐	Eukaryotic cell lines
☒	☐	Palaeontology
☒	☐	Animals and other organisms
☒	☐	Human research participants
☒	☐	Clinical data

Methods

n/a	Involved in the study	
☒	☐	ChIP-seq
☒	☐	Flow cytometry
☒	☐	MRI-based neuroimaging

Antibodies

Antibodies used	Anti-AmCHS IgG, anti-AmCHIL IgG, Anti-His6 antibody
Validation	Details of preparation of the Anti-AmCHS antibody and anti-AmCHIL antibodies are described in the Methods section.<br>Commercial source of Anti-His6 antibody is described in the Methods section.
